# The Yersinia pestis GTPase BipA Promotes Pathogenesis of Primary Pneumonic Plague

**DOI:** 10.1128/IAI.00673-20

**Published:** 2021-01-19

**Authors:** Samantha D. Crane, Srijon K. Banerjee, Kara R. Eichelberger, Richard C. Kurten, William E. Goldman, Roger D. Pechous

**Affiliations:** a Department of Microbiology and Immunology, University of Arkansas for Medical Sciences, Little Rock, Arkansas, USA; b Department of Pediatric Allergy and Immunology, University of Arkansas for Medical Sciences, Little Rock, Arkansas, USA; c Lung Cell Biology Laboratory, Arkansas Children’s Research Institute, Little Rock, Arkansas, USA; d Department of Microbiology and Immunology, University of North Carolina at Chapel Hill, Chapel Hill, North Carolina, USA; University of Pennsylvania

**Keywords:** plague, pneumonic plague, *Yersinia*, *Yersinia pestis*, bacterial GTPases, pathogenesis

## Abstract

Yersinia pestis is a highly virulent pathogen and the causative agent of bubonic, septicemic, and pneumonic plague. Primary pneumonic plague caused by inhalation of respiratory droplets contaminated with Y. pestis is nearly 100% lethal within 4 to 7 days without antibiotic intervention. Pneumonic plague progresses in two phases, beginning with extensive bacterial replication in the lung with minimal host responsiveness, followed by the abrupt onset of a lethal proinflammatory response. The precise mechanisms by which Y. pestis is able to colonize the lung and survive two very distinct disease phases remain largely unknown. To date, a few bacterial virulence factors, including the Ysc type 3 secretion system, are known to contribute to the pathogenesis of primary pneumonic plague. The bacterial GTPase BipA has been shown to regulate expression of virulence factors in a number of Gram-negative bacteria, including Pseudomonas aeruginosa, Escherichia coli, and Salmonella enterica serovar Typhi. However, the role of BipA in Y. pestis has yet to be investigated. Here, we show that BipA is a Y. pestis virulence factor that promotes defense against early neutrophil-mediated bacterial killing in the lung. This work identifies a novel Y. pestis virulence factor and highlights the importance of early bacterial/neutrophil interactions in the lung during primary pneumonic plague.

## Abstract (precis)

Yersinia pestis is a highly virulent pathogen and the causative agent of bubonic, septicemic, and pneumonic plague. Primary pneumonic plague caused by inhalation of respiratory droplets contaminated with Y. pestis is nearly 100% lethal within 4 to 7 days without antibiotic intervention.

## INTRODUCTION

Yersinia pestis is a highly lethal Gram-negative bacterium that causes bubonic, septicemic, and pneumonic plague. Inhalation of aerosols and respiratory droplets contaminated with Y. pestis results in primary pneumonic plague, a rapidly progressing pneumonia that is nearly 100% fatal without antibiotic treatment (1). Due to its ease of transmission by aerosol and potential to cause mass casualties in outbreak and bioterrorism scenarios, Y. pestis is categorized as a Tier 1 Select Agent by the Centers of Disease Control and Prevention (CDC), and a Category A Priority Pathogen by the National Institutes of Health (NIH). While often considered a disease of ancient times, plague continues to be a modern threat, and Y. pestis is endemic on every continent except Antarctica and Australia (2). For example, a 2017 plague epidemic in Madagascar saw approximately 2,600 suspected or confirmed cases of plague, of which roughly 1,700 were pneumonic plague (3).

Essential to Y. pestis virulence is the Ysc type 3 secretion system (T3SS). The *Yersinia* T3SS is a “needle-like” apparatus that directly injects bacterial effector proteins called *Yersinia* outer proteins, or Yops, into the cytosol of target host cells. Once injected, the Yop effectors function to prevent phagocytosis and limit innate immune signaling (4). Aside from the T3SS, few Y. pestis virulence factors have been shown to contribute to disease progression of pneumonic plague. As a result, there is a paucity of information regarding how Y. pestis is able to establish infection and replicate to high numbers in the lung prior to the onset of host inflammatory responses.

BPI-inducible protein A (BipA), also known as TypA, was first identified in Salmonella enterica serovar Typhimurium, and its expression was shown to be induced in the presence of human bactericidal permeability increasing protein (BPI) (5), an antimicrobial peptide (AMP) produced primarily by granulocytes (6, 7). BipA is classified as a translational GTPase with homology to elongation factor G (EF-G) (8, 9) that demonstrates differential ribosomal subunit binding in response to relative guanosine-5′-triphosphate (GTP) and guanosine tetraphosphate (ppGpp) concentrations (10). BipA is highly conserved, and is implicated in regulating virulence mechanisms at the translational level in a number of bacterial species, including Escherichia coli, S. enterica, and Pseudomonas aeruginosa (11). In E. coli, BipA plays a role in K5 capsule production (12), flagella-mediated motility (13, 14), BPI resistance (13), and attachment and effacement to epithelial cells (14). BipA contributes to macrophage uptake and survival in S. enterica serovar Typhi (15), and BPI resistance in S. enterica Typhimurium (16). In P. aeruginosa, BipA is involved in bacterial virulence in amoeba and nematode models, as well as adhesion, biofilm formation, resistance to phagocytosis, antibiotic resistance, and expression of the T3SS (17). While BipA has been shown to contribute to virulence mechanisms in these pathogens, there has been minimal characterization beyond initial *in vitro* analysis. The role of BipA in Y. pestis has yet to be investigated. In this study, we show that deletion of BipA results in decreased bacterial survival upon challenge with human BPI and primary human neutrophils. Further, we show that BipA facilitates bacterial survival in the lung, and contributes to lethality in a murine infection model of pneumonic plague. This work positions BipA as a novel Y. pestis virulence factor that promotes resistance to early neutrophil-mediated bacterial killing in the lung.

## RESULTS

### BipA is involved in the Y. pestis response to challenge with BPI.

Previous work (18) identified 405 bacterial genes that were differentially expressed in Y. pestis isolated from the murine lung during infection compared to broth-grown bacteria. Of these, the gene *bipA* (or *YPO0026*) encoding the bacterial translational GTPase BipA was upregulated 2.3-fold during pneumonic plague. Expression of BipA is known to be induced in the presence of BPI (5), an AMP released in primary granules by neutrophils (19–21). BipA, in turn, has been implicated in regulating expression of proteins, including known virulence factors, in a number of pathogens (8, 9, 11, 14, 17, 22–25). We sought to determine if the absence of BipA impacted expression of BPI-induced proteins in Y. pestis. We generated a strain of Y. pestis CO92 lacking *bipA* (Δ*bipA*) and evaluated total protein expression by liquid chromatography-tandem mass spectrometry (LC-MS/MS) in wild type and Δ*bipA* strains in the logarithmic phase of broth-grown culture and during incubation with BPI. This analysis revealed a number of proteins that were differentially expressed (at least 2-fold difference) in Δ*bipA* Y. pestis compared to wild-type Y. pestis under both conditions (Table S1 and S2 in the supplemental material), as well as altered regulation of an additional set of proteins in the presence of BPI. For example, we observed increased expression of the zinc import ATP-binding protein ZnuC and decreased expression of Der, putative type 6 secretion system components, and various uncharacterized proteins in Δ*bipA* Y. pestis compared to wild-type Y. pestis under both conditions. During incubation with BPI, we saw differential regulation of additional proteins, including ClpB, YscJ, and a number of other uncharacterized proteins. Deletion of BipA did not appear to effect transcription of *znuC*, *YPO0502*, *psaA*, and *yopH*, each of which encode proteins that showed altered expression in the absence of BipA (Fig. S1). Similar to its function in other bacteria, our data suggest that BipA contributes to the bacterial response to challenge with BPI.

**FIG 1:**
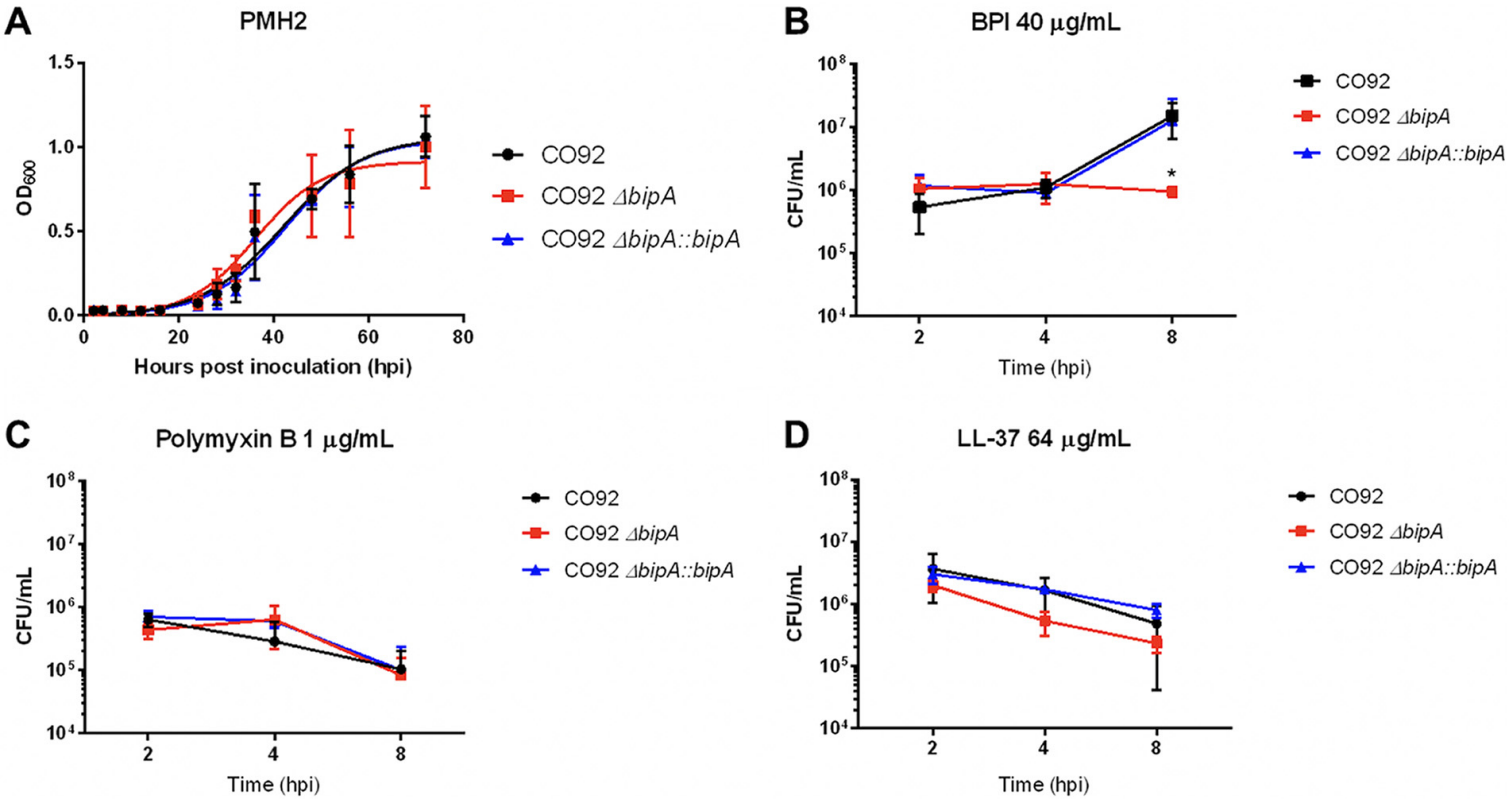
BipA is involved in the Y. pestis response to challenge with BPI. (A to D) Wild-type, Δ*bipA*, and Δ*bipA*::*bipA* Y. pestis CO92 from overnight culture grown at 37°C in BHI medium was diluted into complete PMH2 medium and optical density at 600 nm (OD_600_) was measured at the indicated time points to construct a growth curve. Wild-type, Δ*bipA*, and Δ*bipA*::*bipA* Y. pestis CO92 were inoculated into PMH2 medium containing 40 μg/ml BPI (B), 1 μg/ml polymyxin B (C), or 64 μg/ml LL-37 (D) and at each time point CFU/ml was enumerated by standard plate count. Significance was calculated with two-way ANOVA; *, *P* ≤ 0.05. Error bars represent standard deviation (SD). Data are presented as a pool of three independent experiments.

BPI is released from the granules of neutrophils and has high affinity for the lipid A component of bacterial lipopolysaccharide (LPS) (19). Binding of BPI to LPS inhibits the endotoxic activity of LPS, facilitates opsonization of bacteria, and has potent bactericidal activity (6, 26, 27). BipA has been shown to contribute to bacterial resistance to BPI in E. coli and S. enterica Typhi (13, 16). We sought to determine if BipA contributes to Y. pestis resistance to BPI. To this end, we incubated wild-type Y. pestis strain CO92, Δ*bipA*, or the Δ*bipA*::*bipA* complement strain with a sub-MIC of BPI (40 μg/ml) at 37°C for 8 h, and enumerated CFU of each strain at 2, 4, and 8 h postinoculation (hpi). While deletion of *bipA* had no impact on Y. pestis growth in minimal medium (Fig. 1A), we observed a significant decrease in survival of Y. pestis lacking BipA by 8 hpi compared to wild-type and BipA-complemented Y. pestis, indicating a role for BipA in bacterial resistance to BPI (Fig. 1B). To determine if Δ*bipA* was attenuated in the presence of antimicrobial peptides in general, we also evaluated bacterial survival upon challenge with polymyxin B and LL-37. Deletion of *bipA* did not impact Y. pestis sensitivity to sub-MICs of polymyxin B or LL-37, suggesting that BipA may not contribute to resistance to all AMPs (Fig. 1C and D). Evaluating the MIC of each antimicrobial peptide for all three strains showed no difference in AMP resistance (Table S3). These results indicate that BipA contributes to the bacterial response to challenge with BPI.

### BipA contributes to resistance to neutrophil-mediated bacterial killing.

**FIG 2:**
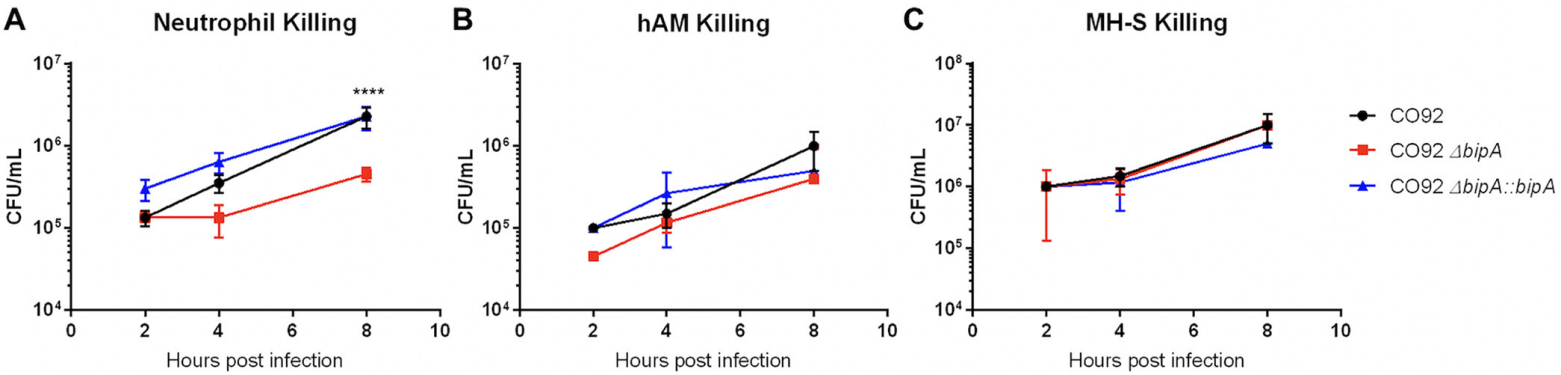
BipA contributes to resistance to neutrophil-mediated bacterial killing. (A to C) Human primary neutrophils (A), human primary alveolar macrophages (hAM) (B), and immortalized murine alveolar macrophages (MH-S) (C) were inoculated with wild-type, Δ*bipA*, and Δ*bipA*::*bipA* Y. pestis CO92 using an MOI of 1:1 bacteria to host cells. Surviving bacteria counts were determined at 2, 4, and 8 hpi by serial dilution and standard plate counting. Significance was calculated with two-way ANOVA; ****, *P* ≤ 0.0001. Data are representative of three independent experiments.

BPI is primarily produced by granulocytes such as neutrophils (6). We therefore sought to determine whether BipA contributes to bacterial defense against neutrophils. We inoculated primary human neutrophils with wild-type, Δ*bipA*, or Δ*bipA*::*bipA* Y. pestis CO92, and monitored bacterial survival for 8 h. We observed significantly decreased survival of Δ*bipA* Y. pestis compared to wild-type and Δ*bipA*::*bipA* Y. pestis in the presence of primary human neutrophils (Fig. 2A), indicating that BipA contributes to bacterial resistance to neutrophil-mediated bacterial killing *in vitro*. To determine if BipA contributes to resistance to killing by professional phagocytes in general, we also evaluated bacterial survival during incubation with primary human alveolar macrophages (hAM) and immortalized murine alveolar macrophages (MH-S) (Fig. 2B and C). Y. pestis encounters alveolar macrophages early during infection, and these initial host/pathogen interactions are important to establishing infection in the lung. We did not observe a significant difference in Δ*bipA* survival compared to wild type or complemented Δ*bipA* during incubation with macrophages, suggesting that the contribution of BipA to bacterial survival may be specific to neutrophils. Gentamicin protection assays also revealed no difference in the ability of primary human neutrophils or MH-S cells to phagocytose wild-type, Δ*bipA*, or Δ*bipA*::*bipA* Y. pestis strains (Fig. S2).

**FIG 3:**
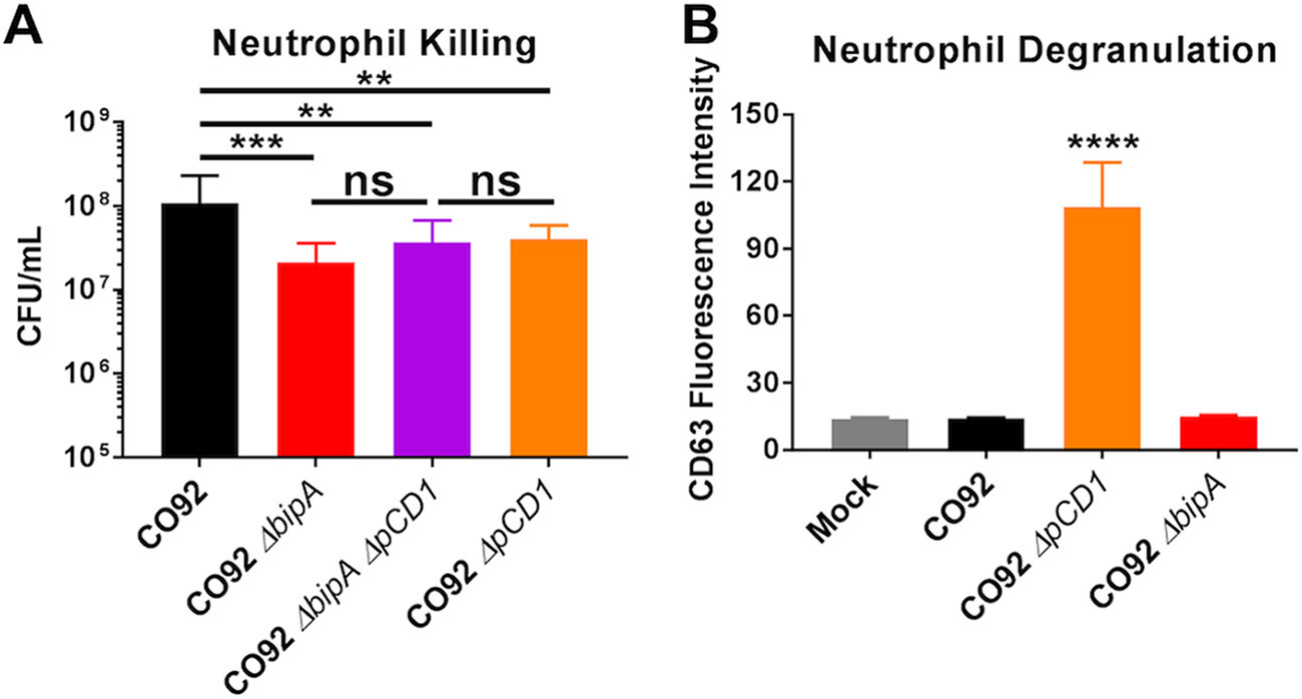
Role of the T3SS in BipA-mediated resistance to neutrophils. (A) Human primary neutrophils were inoculated with wild-type, Δ*bipA*, Δ*pCD1*, or Δ*bipA* Δ*pCD1* Y. pestis CO92 at an MOI of 1:1 bacteria to host cells. Surviving bacteria counts were determined at 2, 4, and 8 hpi by serial dilution and plating. Significance was calculated using two-way ANOVA; ns, no significant difference; **, *P* ≤ 0.005; ***, *P* ≤ 0.0005. (B) Human primary neutrophils were inoculated with wild-type, Δ*bipA*, or Δ*pCD1* Y. pestis CO92 using an MOI of 10:1 bacterial to host cells for 1 h at 37°C. CD63 mean fluorescence intensity was measured by flow cytometry. Significance relative to mock-inoculated neutrophils was calculated using one-way ANOVA with Tukey’s multiple-comparison test; ****, *P* ≤ 0.0001. For both experiments, error bars represent SD. Data are presented as pool of at least two independent experiments.

The Yersinia pestis T3SS (encoded on the pCD1 plasmid) inhibits phagocytosis of bacteria, limits innate immune signaling (4, 28), and is essential for virulence. The *Yersinia* T3SS is critical for defense against phagocytosis by neutrophils (29–31). BipA was shown to regulate expression of the T3SS of P. aeruginosa (17), indicating that BipA-related phenotypes may result from the inability to effectively target host cells for T3SS. Though we did not see major differences in expression of the T3SS in the absence of BipA, we still sought to determine if reduced T3SS could explain increased Δ*bipA* Y. pestis sensitivity to killing by primary human neutrophils. Initial efforts to quantify T3SS in primary neutrophils were unsuccessful, likely due to the rapid turnover and sensitivity of primary neutrophils to manipulation in culture. As an alternative approach, we incubated primary human neutrophils with wild-type, Δ*bipA*, Δ*bipA*::*bipA*, Δ*pCD1* lacking the T3SS, or Δ*bipA* Δ*pCD1* Y. pestis CO92 and monitored bacterial survival. We observed a similar decrease in the viability of Δ*pCD1* and Δ*bipA* Δ*pCD1* Y. pestis compared to the Δ*bipA* strain in the presence of neutrophils (Fig. 3A). This finding does not confirm a link between BipA and T3SS, but does indicate it is possible that BipA-mediated resistance to neutrophil-mediated killing is due to its regulation of the T3SS. To investigate this further, we sought to determine if the absence of BipA impacted the effects of the T3SS on target neutrophils. The Y. pestis T3SS was recently shown to inhibit degranulation of neutrophils via the combined activity of YopE and YopH (30, 32). We therefore investigated the ability of Y. pestis Δ*bipA* to inhibit neutrophil degranulation compared to wild-type Y. pestis as a readout of T3SS. Primary human neutrophils were inoculated with wild-type, Δ*bipA*, or Δ*pCD1* Y. pestis using an MOI of 10:1 bacteria to host cells, and neutrophil degranulation was evaluated by flow cytometric detection of CD63-PE-Cy7 as a marker of primary granule release (21). We observed no difference in CD63 fluorescence intensity between wild-type and Δ*bipA* Y. pestis, indicating that loss of BipA did not impact the ability of Y. pestis to inhibit neutrophil degranulation by the T3SS (Fig. 3B). As expected, we observed increased CD63 fluorescence intensity consistent with neutrophil degranulation after incubation with Δ*pCD1* Y. pestis lacking the T3SS effectors and machinery. Gentamicin protection assays also showed similar resistance of Δ*bipA* Y. pestis (compared to both wild-type and BipA-complemented Y. pestis) to phagocytosis by neutrophils (Fig. S2), suggesting similar T3SS function. Together, these data show that BipA likely promotes Y. pestis resistance to neutrophil-mediated killing via a T3SS-independent mechanism.

### BipA promotes Y. pestis virulence and bacterial survival in a murine model of primary pneumonic plague.

**FIG 4:**
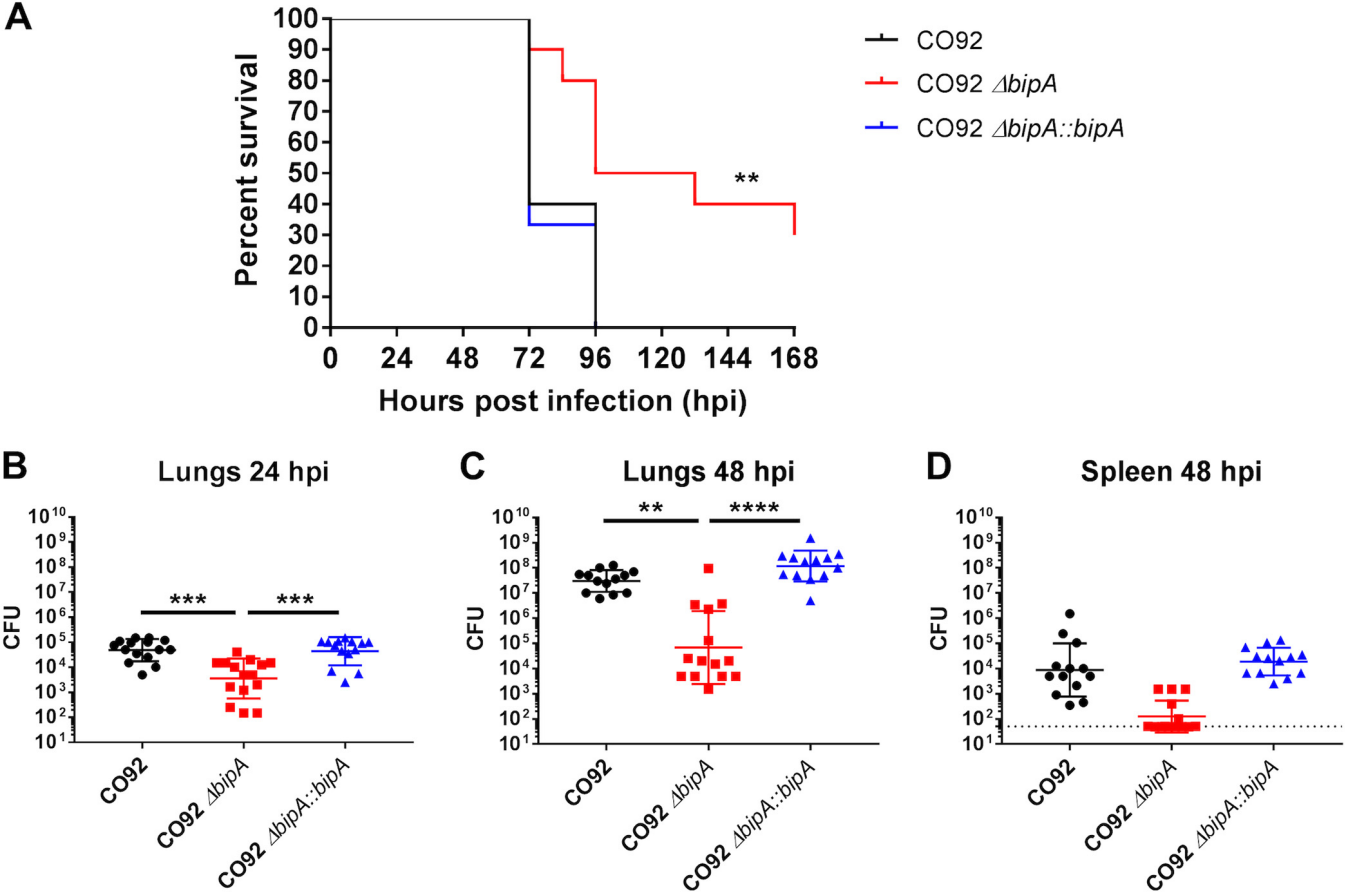
BipA promotes Y. pestis virulence and bacterial survival in a murine model of primary pneumonic plague. (A) C57BL/6 female mice (*n* = 10 per group) were infected with 10^3^ CFU of either wild-type CO92, Δ*bipA*, or Δ*bipA*::*bipA* Y. pestis via the intranasal route. Survival over a period of 7 days was recorded. Significance was calculated using a log rank test; **, *P* ≤ 0.005. Bacterial burdens (*n* = 13 to 15 per group) in the lung (B and C) and spleen (D) were determined at 24 h (lung only) and 48 h postinfection by plating serial dilutions of organ homogenates on BHI agar plates. Significance was calculated using one-way ANOVA; **, *P* ≤ 0.005; ***, *P* ≤ 0.0005; ****, *P* ≤ 0.0001. Error bars represent SD. Data in panel A are presented as representative of three independent experiments. Data in panels B to D are pooled from three independent experiments.

We next sought to determine if BipA contributes to virulence *in vivo* during primary pneumonic plague. Groups of 4- to 6-week-old C57BL/6 female mice were inoculated via the intranasal route with 10^3^ CFU of wild-type, Δ*bipA*, or Δ*bipA*::*bipA* Y. pestis and survival was observed over time. Mice inoculated with Δ*bipA* Y. pestis showed increased survival compared to mice inoculated with wild-type and Δ*bipA*::*bipA* Y. pestis (Fig. 4A). To determine if BipA contributes to bacterial survival in the lung and/or dissemination to the spleen, bacterial burdens were quantified at 24 hpi (lungs only) and 48 hpi (lungs and spleen). While deletion of *bipA* did not impact bacterial growth in culture (Fig. 1A), animals inoculated with Δ*bipA* Y. pestis had significantly decreased bacterial burdens in the lung compared to animals infected with wild-type or BipA-complemented Y. pestis strains at both 24 and 48 hpi (Fig. 4B and C). While we did not observe a statistically significant difference in Δ*bipA* Y. pestis bacterial burdens in the spleen (Fig. 4D) compared to wild-type and Δ*bipA*::*bipA* Y. pestis-infected animals, we failed to detect measurable burdens in 6 out of 15 Δ*bipA* Y. pestis-infected animals, indicating a lack of bacterial dissemination from the lung. These data show that BipA promotes Y. pestis virulence in a murine model of primary pneumonic plague and contributes to bacterial survival in the lung during infection. Importantly, decreased survival of the bacterium was evident at 24 hpi, indicating that BipA likely contributes to early events facilitating survival of Y. pestis in the lung.

**FIG 5:**
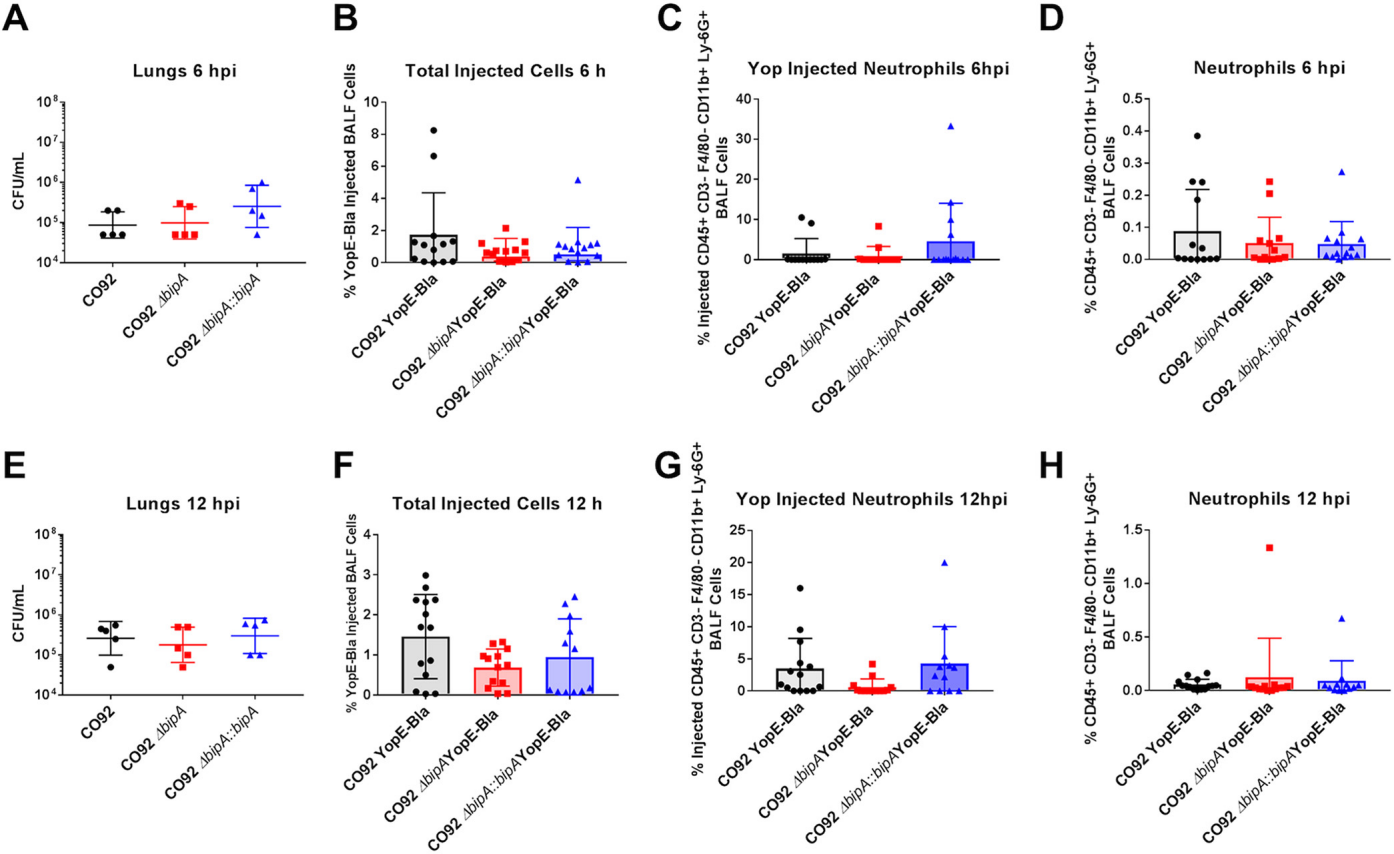
BipA does not impact Y. pestis T3SS *in vivo* at 6 and 12 hpi. (A to H) C57BL/6 female mice (*n* = 5 per group) were infected with 5 × 10^4^ CFU of either wild-type CO92, Δ*bipA*, or Δ*bipA*::*bipA* Y. pestis via the intranasal route, and infected lungs were analyzed at 6 (A to D) and 12 (E to H) hpi. Bacterial burdens were determined at 6 (A) and 12 (E) hpi by plating serial dilutions of organ homogenates on BHI agar plates. C57BL/6 mice (*n* = 12 to 14 per group) were infected with 5 × 10^4^ CFU of either wild-type (YopE-Bla) CO92, Δ*bipA* YopE-Bla, or Δ*bipA*::*bipA* YopE-Bla Y. pestis via the intranasal route. The proportions of YopE-injected bronchoalveolar lavage fluid (BALF) cells were determined via flow cytometry at 6 (B) and 12 (F) hpi. The proportion of YopE-injected neutrophils (CD45^+^ CD3^−^ F4/80^−^ CD11b^+^ Ly-6G^+^) were determined via flow cytometry at 6 (C) and 12 (G) hpi. The proportion of neutrophils in the BALF was determined via flow cytometry at 6 (D) and 12 (H) hpi. Significance was calculated using one-way ANOVA; **, *P* ≤ 0.005. Error bars represent SD. Data are presented as a pool of at least two independent experiments.

**FIG 6:**
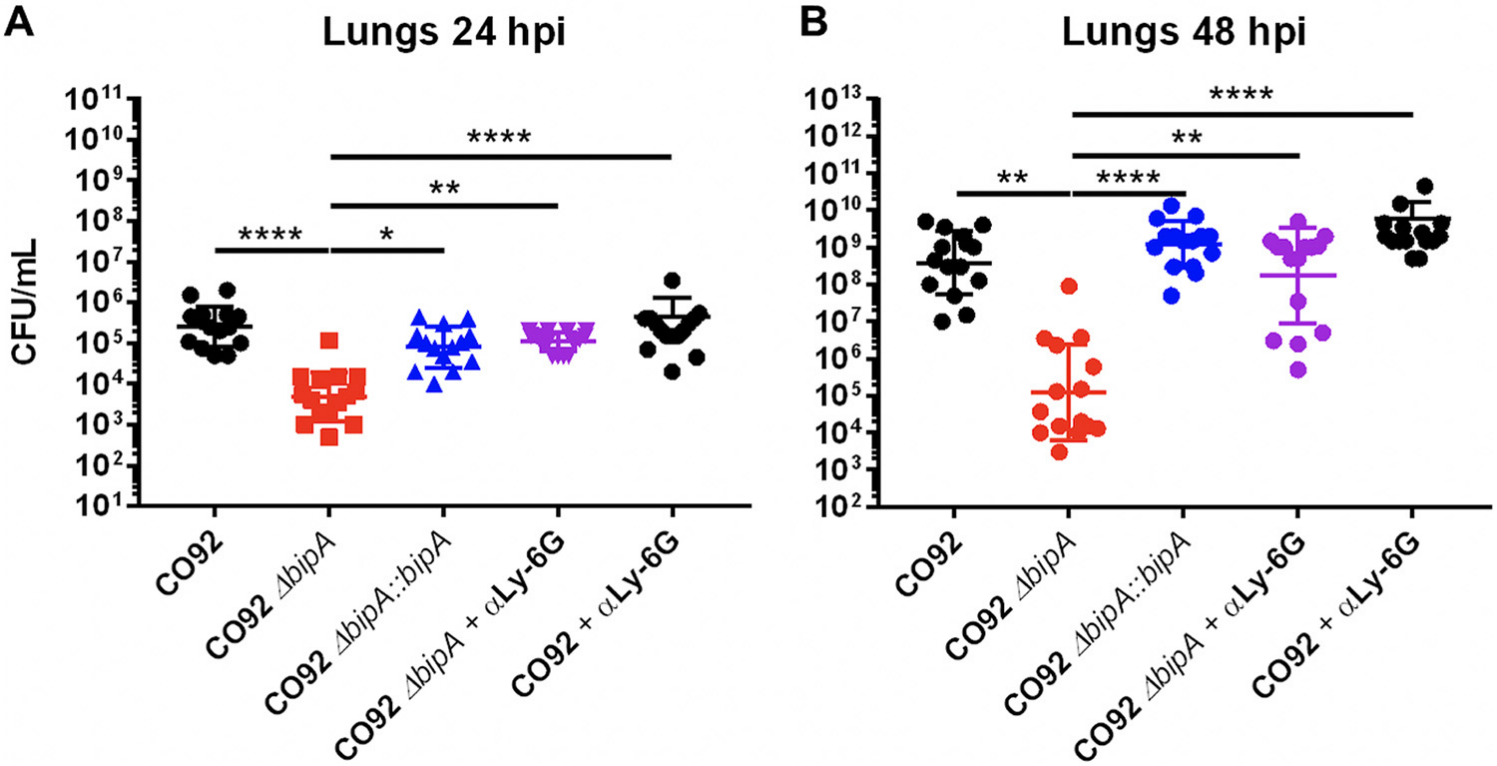
Neutrophils control Δ*bipA* Y. pestis *in vivo*. C57BL/6 mice (*n* = 14 to 15 per group) were infected with 10^3^ CFU of either wild-type CO92 or Δ*bipA* Y. pestis via the intranasal route. αLy-6G-treated mice were given anti-mouse Ly-6G antibody via tail vein injection at one day prior to infection and on the day of infection. Bacterial burdens in the lung were determined at 24 (A) and 48 (B) hpi by homogenization, serial dilution, and plating. Significance was calculated using two-way ANOVA; **, *P* ≤ 0.005; ***, *P* ≤ 0.0005; ****, *P* ≤ 0.0001. Error bars represent SD. Data are presented as a pool of three independent experiments.

Our *in vitro* data suggest that BipA contributes to bacterial resistance to neutrophil-mediated killing via a mechanism independent of regulation of the T3SS. To determine if this is the case *in vivo*, we evaluated the T3SS in the lungs at 6 and 12 hpi, when bacterial burdens were roughly equivalent between wild-type and Δ*bipA* strains (Fig. 5A and E). To this end, C57BL/6 mice were inoculated with 5 × 10^4^ CFU of wild-type, Δ*bipA*, or Δ*bipA*::*bipA* Y. pestis YopE-Bla reporter strains. These strains express wild-type YopE as well as a protein consisting of the first 100 amino acids of YopE fused to a truncated TEM β-lactamase gene (*bla*), and have been used extensively to detect the T3SS *in vitro* and *in vivo* (33–35). Upon incubation of infected cells with the fluorescent Bla substrate CCF2-AM, mammalian cells can be differentiated as Yop-injected (blue fluorescence) or uninjected (green fluorescence). At 6 and 12 hpi, there were no significant differences in levels of Yop-injected cells in the bronchoalveolar lavage fluid (BALF) between wild-type, Δ*bipA*, or Δ*bipA*::*bipA* Y. pestis infections (Fig. 6B and F). As BipA contributes to bacterial survival upon challenge with neutrophils *in vitro*, we quantified T3SS into neutrophils specifically. We observed no differences in Δ*bipA* Y. pestis Yop injection into neutrophils at 6 hpi or 12 hpi compared to wild-type and BipA-complemented Y. pestis (Fig. 5C and G). No difference in neutrophil influx into BALF was observed at 6 or 12 hpi with any strain (Fig. 5D and H). Together, these data indicate that BipA likely does not contribute significantly to the Y. pestis T3SS *in vivo*.

### Neutrophils are responsible for limiting growth of Y. pestis lacking BipA.

The finding that Y. pestis lacking BipA was more sensitive to neutrophil-mediated killing *in vitro* prompted us to investigate whether BipA promotes bacterial resistance to neutrophils *in vivo*. To test this, groups of mice were inoculated with 10^3^ CFU of wild-type, Δ*bipA*, or Δ*bipA*::*bipA* Y. pestis CO92 and bacterial burdens were analyzed in the lung at 24 and 48 hpi. One cohort of Δ*bipA-*infected mice received 20 μg of anti-mouse Ly-6G antibody (αLy-6G) prior to and at the time of infection to deplete neutrophils (Fig. 6). At both 24 (Fig. 6A) and 48 hpi (Fig. 6B), depletion of neutrophils resulted in complete restoration of Δ*bipA* Y. pestis growth in the lung. Neutrophil depletion did not significantly enhance bacterial burdens of wild-type Y. pestis at 24 hpi or 48 hpi compared to untreated mice, indicating that Δ*bipA* Y. pestis is exceptionally susceptible to neutrophil-mediated killing *in vivo*. These results indicate that neutrophils control Δ*bipA* Y. pestis growth in the lung, and that BipA contributes to bacterial resistance to neutrophil-mediated killing.

## DISCUSSION

Y. pestis employs a potent arsenal of virulence factors to cause multiple manifestations of plague. While many virulence factors have been characterized, only a few have been shown to contribute to the pathogenesis of pneumonic plague specifically. The precise mechanisms underlying biphasic disease progression of pneumonic plague are unknown, and it is likely that a number of bacterial and host mediators of pathogenesis are yet to be identified. Antibiotic-resistant strains of Y. pestis have emerged in plague-endemic foci (36), warranting investigation of putative virulence factors that contribute to disease and might be exploited therapeutically. In this study, we investigated the role of a novel virulence factor, BipA, in the pathogenesis of primary pneumonic plague. We show that BipA confers resistance to BPI *in vitro* and contributes to bacterial resistance to neutrophil-mediated killing, likely via a T3SS-independent mechanism. We further demonstrate that BipA promotes Y. pestis survival in lungs and bacterial dissemination to the spleen. Importantly, depletion of neutrophils rescues growth of the Δ*bipA* Y. pestis strain in the lung, confirming the importance of early bacterial-neutrophil interactions in the lung. This is the first time BipA has been evaluated in *Yersinia*, and to our knowledge is the first study to demonstrate the importance of BipA in bacterial virulence *in vivo* using a murine model of infection.

There is little known about the precise role BipA plays in bacterial translation or virulence. Studies from multiple labs using E. coli in low temperature conditions have shown that BipA promotes efficient assembly of ribosomal proteins (22, 25). Furthermore, BipA is shown to have binding capabilities to the ribosome and 30S small ribosomal subunit in response to the relative abundance of GTP and ppGpp (9, 10, 23), suggesting a role in bacterial amino acid starvation responses (37). Other work used *in vitro* approaches to show that BipA has a role in regulating critical bacterial virulence mechanisms such as T3SS, adhesion, AMP resistance, motility, capsule expression, and survival and uptake into macrophages (13–15, 17). Further work is needed to determine specifically how BipA contributes to stress responses at the mammalian body temperature and changes in protein expression that we observed here in response to challenge with BPI.

Our data indicate that the presence of BipA may impact the translation of a myriad of proteins in response to BPI, and potentially *in vivo*. These include known proteins such as Der and ZnuC. Der (also known as YphC in Bacillus subtilis) is a tandem GTPase functioning in 50S ribosomal subunit biogenesis and is essential for growth in E. coli (38, 39). Our proteomic analysis showed a decrease in Der abundance in Y. pestis lacking BipA. ZnuC is the ATPase component of the zinc ion ATP-binding cassette (ABC) transporter system encoded by *znuABC*. Our proteomic analysis also suggested that the presence of BipA impacts the expression of a number of hypothetical proteins, including predicted type 6 secretion system (T6SS) components. The Hcp-like component encoded by *YPO0502* is implicated in autoagglutination (40) and has decreased expression in Δ*bipA* Y. pestis upon BPI challenge, according to our proteomic analysis. The precise roles of this and other predicted T6SS components in *Yersinia* have yet to be elucidated, but studies have suggested their roles in environment adaptation (41) and virulence (42). While it is unclear if BipA directly impacts translation of specific proteins, dysregulated expression of important virulence factors and metabolic proteins in its absence likely contribute to decreased viability in the presence of neutrophils and BPI, and may explain the decreased fitness of Δ*bipA* Y. pestis *in vitro* and *in vivo*.

The early stage of pneumonic plague has been described as “preinflammatory,” characterized by minimal host responsiveness despite rapid outgrowth of Y. pestis in the lung (43). The Ysc T3SS is essential for colonizing and maintaining infection in the lung, as it functions to inhibit phagocytosis (4) and various immune signaling processes (44) by primarily targeting alveolar macrophages in the lung early in infection (35). This interaction is further facilitated by adhesins such as Ail, Psa, and Pla that encourage close contact between Y. pestis and host cells targeted for T3SS (45–47). The resulting effect is the creation of localized pockets of immunosuppression. Despite this, it is becoming clear from our lab and others that an early wave of neutrophils can be detected within the first 24 h of pulmonary infection (35, 48) and Y. pestis must overcome this early onslaught. While several virulence factors have been identified and characterized in the later “proinflammatory” stage of pneumonic plague, where increased proinflammatory cytokines are expressed and increased immune cell infiltration occurs (43), it is unclear which play roles in establishing Y. pestis pulmonary infection. Here, we pose BipA as a novel virulence factor that aids in establishing Y. pestis pulmonary infection by contributing to resistance to early neutrophil infiltration into the lungs.

In summary, this work characterizes the role of the Y. pestis translational GTPase BipA in primary pneumonic plague. We show that BipA promotes resistance to neutrophil-mediated killing in a T3SS-independent manner. This effect is likely indirect, due to the impact of BipA on the expression of a number of proteins, including previously identified virulence factors, metabolic genes, and uncharacterized proteins in the face of host challenges during pulmonary infection. Future work will focus on identifying the mechanism of BipA-mediated protein expression and determining how BipA mediates resistance to neutrophil-mediated bacterial killing.

## MATERIALS AND METHODS

### Bacterial strains.

Y. pestis strain CO92, CO92 Δ*bipA*, and CO92 Δ*pCD1* were obtained from the lab of William Goldman (UNC-Chapel Hill). The Δ*bipA* Y. pestis strain was generated using a modified lambda red recombination described previously (49). Upstream and downstream sequences of *bipA* were amplified by PCR using the following primers: 5′-TTACTGCATTTATGGTGTTCAGGCATT-3′, 5′-GAAGCAGCTCCAGCCTACACCACAGCTTTTTTGCCTCAGGCATTTAG-3′ (upstream forward and reverse, respectively); 5′-GGTCGACGGATCCCCGGAATTTCTTCTTTGCATTGTTGATACTTAGGGC-3′, and 5′-CTGCTCGAAACTCAAGGATATCCC-3′ (downstream forward and reverse, respectively. Underlined sequence indicates tags complementary to sequence tags added to primers used to amplify a kanamycin resistance marker. PCR product of sequence immediately upstream and downstream of the target loci was then used with the kanamycin resistance cassette as the template for splicing by overlap extension PCR to generate a PCR fragment for allelic exchange. The CO92 Δ*bipA*::*bipA* Y. pestis strain was generated as described previously (49) using Tn7-based integration (50) of *bipA* into the Y. pestis chromosomal *glmS-pstS* region. The following primers were used to amplify the *bipA* open reading frame: 5′-TTGACCTGTTCGTTA-3′ and 5′-ACCGCTTTACCCTGA-3′ (forward and reverse primers, respectively). CO92 Δ*bipA* YopE-Bla, CO92 Δ*bipA*::*bipA* YopE-Bla, and CO92 Δ*pgm* Δ*bipA* Δ*bipA*::*bipA* YopE-Bla strains were generated as described previously (35, 51). Y. pestis strains were grown on brain heart infusion (BHI) agar (Difco) at 26°C for 2 to 3 days. Strains were confirmed by growth on Congo red plates, and PCR was performed to confirm the presence of the *pgm* locus and the pCD1 plasmid for wild-type and mutant strains. For infection, Y. pestis strains were inoculated into 10 ml BHI broth supplemented with 2.5 mM CaCl_2_ and incubated at 37°C for 12 to 16 h with constant shaking. Infections with fully virulent Y. pestis CO92 were performed in the UAMS Biosafety level 3 (BSL-3) facility. For bacterial growth curve analysis, optical density of Y. pestis cultured in BHI or PMH2 medium was obtained using a spectrophotometer at the indicated time points.

### Cell culture and cell culture infections.

MH-S cells were obtained from ATCC and were cultured in RPMI 1640 (Corning) medium containing 10% fetal bovine serum (FBS) (Gibco) and Antibiotic-Antimycotic (Gibco). Cell cultures were incubated at 37°C with 5% CO_2_. To perform bacterial killing assays, primary human neutrophils (Astarte Biologics), primary human alveolar macrophages (hAMs), and MH-S cells were cultured briefly in IMDM (Gibco), DMEM:F12K 1:1 (Corning), or RPMI 1640 (Corning) medium supplemented with 10% human AB sera or FBS. After determining cell density, cells were inoculated with Y. pestis using an MOI of 1:1 CFU bacteria to host cell. Cells were then incubated at 37°C with 5% CO_2_ for up to 8 h. At 2, 4, and 8 hpi, cells were scraped and 100 μl from each technical replicate was serially diluted and plated on BHI agar to determine CFU. Gentamicin protection assays were performed by inoculating cells with Y. pestis strains using an MOI of 1:1. After the indicated time points postinfection, cells were treated with 8 μg/ml gentamicin-containing (52) cell culture medium for 1 h. After gentamicin treatment, medium was aspirated from cells and replaced with sterile diH_2_O to lyse cells. Technical replicates were serially diluted and plated on BHI agar to determine levels of intracellular Y. pestis.

### Antimicrobial peptide susceptibility tests.

To determine the MIC of BPI, polymyxin B, and LL-37 needed to inhibit growth of Y. pestis strains, strains were cultured for 16 h in BHI broth supplemented with 2.5 mM CaCl_2_ at 37°C. Serial dilutions of BPI, polymyxin B, and LL-37 were prepared in 96-well plates containing 100 μl BHI and 2.5 mM CaCl_2_ per well. Aliquots of 10^5^ CFU of wild-type (CO92), Δ*bipA*, or Δ*bipA*::*bipA* Y. pestis were inoculated into wells and incubated overnight at 37°C with shaking. The MIC was determined the following day by assessing the concentration of antimicrobial peptide needed to inhibit Y. pestis growth. To determine susceptibility to subinhibitory concentrations of antimicrobial peptide, Y. pestis strains were cultured for 16 h in BHI broth and then aliquots of wild-type, Δ*bipA*, and Δ*bipA*::*bipA* Y. pestis were subcultured in PMH2 (53) medium with or without 40 μg/ml BPI (R&D Systems), 1 μg/ml polymyxin B (Fisher), or 64 μg/ml LL-37 (Tocris Biosciences). Cultures were grown at 37°C with constant shaking and at 2, 4, and 8 hpi, 100 μl from each technical replicate was serially diluted and plated on BHI agar to determine CFU.

### Preparation of human alveolar macrophages.

To isolate human alveolar macrophages (hAMs), lungs obtained from anonymous donors through the Arkansas Organ Recovery Agency were lavaged using phosphate-buffered saline (PBS). Lavage fluid was centrifuged at 500 × *g* for 10 min and treated with red blood cell lysis buffer (0.15 M NH_4_Cl, 12 mM NaHCO_3_, and 0.1 mM EDTA) for 5 min. The remaining cells were washed with PBS and reconstituted in DMEM-F12K 1:1 (GE) supplemented with 10% FBS (Gibco) and Antibiotic-Antimycotic (Gibco). Cells were plated in 12-well plates and incubated at 37°C with 5% CO_2_ for 4 to 5 days. Cells were counted prior to infection.

### Animals, animal infections, and ethical approval.

All animal experiments were conducted with approval from the UAMS Institutional Animal Ethics Committee. Six- to 8-week old C57BL/6J female mice were obtained from Jackson Laboratories. Mice were provided with food and water *ad libitum* and maintained at 25 to 26°C with 40 to 70% humidity. To prepare mice for infection, mice were anesthetized using ketamine/xylazine injected via the intraperitoneal route. Once anesthetized, mice were inoculated via the intranasal route with lethal doses (10^3^ bacteria) of Y. pestis delivered in 20 μl PBS for survival experiments. For YopE secretion analysis, mice were inoculated via intranasal route with 5 × 10^4^ CFU Y. pestis. Mice were sacrificed using a lethal dose of sodium pentobarbital injected via the intraperitoneal route. To determine bacterial burdens in lungs and spleens of mice, lungs and spleens were homogenized at 24 h (lungs only) and 48 h postinfection. Organ homogenates were serially diluted and plated on BHI agar to determine CFU. For neutrophil depletion, 20 μg Ly-6G antibody (clone 1A8, BioLegend) was injected via the intravenous route one day prior to and on the day of infection, as described previously (48).

### Neutrophil degranulation assay.

Neutrophils were isolated from human peripheral blood samples and degranulation was quantified as described previously (30). Briefly, 10^6^ isolated human neutrophils were infected in triplicate with 10^7^ CFU of wild-type, Δ*bipA*, or Δ*pCD1* Y. pestis CO92 or mock-inoculated with PBS. Neutrophils and Y. pestis were incubated for 1 h at 37°C. After incubation, neutrophils were washed in PBS with 2% FBS, then stained for 25 min at 4°C with CD63-phycoerythrin-Cy7 (clone H5C6, Invitrogen) as a marker of primary granule exocytosis (21). Neutrophils were washed once in 2% FBS in PBS containing propidium idodide (Invitrogen) to exclude dead cells from analysis. Neutrophils were acquired using a Millipore Guava 6HT flow cytometer and analyzed with the InCyte EasyCyte v3.1 software.

### Preparation of BALF for flow cytometry.

Bronchoalveolar lavage fluid (BALF) was retrieved from mice postmortem. BALF was centrifuged for 5 min at 500 × *g* to pellet cells. Cells were resuspended in 3% FBS in PBS containing the following antibodies (1:500 dilution) for 30 min at 4°C to stain cell surface markers: CD45-phycoerthrin (clone 30-F-11, BD Biosciences), CD3-allophycocyanin-Cy7 (clone 17A2, BD Biosciences), CD11b-Alexa Fluor 700 (clone M1/70, BD Biosciences), CD11c-Brilliant Violet 786 (clone HL3, BD Biosciences), F4/80-allophycocyanin (clone BM8, Invitrogen), and Ly-6G-phycoerthrin-Cy7 (clone 1A8, BD Biosciences). After antibody staining, cells were centrifuged at 500 × *g* for 5 min and the supernatant was removed. Cells were subsequently stained using 1× CCF2-AM (Invitrogen) for 30 min at room temperature. Cells were pelleted at 500 × *g* for 5 min and resuspended in 2% formalin in PBS for fixation for 20 min at room temperature. After fixation, cells were pelleted and resuspended in 200 μl of 3% FBS in PBS for flow cytometry. The following cell populations were identified as described previously (35): alveolar macrophages (F4/80^+^CD11b^mid/low^CD11c^high^), CD11b^high^ interstitial/exudate macrophages (F4/80^+^CD11b^high^CD11c^low/mid^), F4/80^+^CD11b^high^CD11c^high^ macrophages, monocytes (F4/80^−^CD11b^high^CD11c^low^Ly-6G^−^), CD11b^high^ and CD11b^low^dendritic cells (F4/80^−^CD11c^high^CD11b^high or low^), and neutrophils (F4/80^−^CD11c^low^CD11b^high^Ly-6G^+^).

### Proteomic analysis.

To obtain protein extracts from broth-grown Y. pestis, equal optical densities of Y. pestis CO92 and Y. pestis CO92 Δ*bipA* cultures were spun down at 10,000 × *g* for 5 min to pellet bacterial cells. To obtain bacterial protein extracts from Y. pestis incubated with BPI, Y. pestis was incubated with 40 μg/ml BPI or PBS for 30 min at 37°C. Bacteria were spun at 10,000 × *g* for 5 min to pellet bacteria. Bacterial pellets were resuspended in 1× Laemmeli buffer (Bio-Rad) and boiled for 5 min at 96°C to lyse cells. Volumes of each cell lysate sample (normalized to CFU) were resolved via SDS-PAGE and stained with Coomassie brilliant blue to visualize protein bands. Protein gels were subsequently submitted to the UAMS Proteomics Core for in-gel trypsin digestion, MS/MS analysis, and protein identification and quantification. Spectral counts were exported from Scaffold (Proteome Software) to Microsoft Excel for further statistical analyses.

### Statistical analyses.

Statistical analyses were done using one-way and two-way analysis of variance (ANOVA) and log rank test where applicable. *P* values of <0.05 are represented as *, of <0.005 as **, of <0.0005 as ***, and of <0.0001 as ****. All statistical analyses were performed using GraphPad Prism v7.04 software.

## Supplementary Material

Supplemental file 1

Supplemental file 2

Supplemental file 3

Supplemental file 4

Supplemental file 5
