# Supplementary material for: The Yersinia pestis GTPase BipA Promotes Pathogenesis of Primary Pneumonic Plague
Source: Infect Immun. 2021 Jan 19;89(2):e00673-20. doi: 10.1128/IAI.00673-20 (PMC7822129; doi:10.1128/IAI.00673-20)
Supplement: Supplemental file 1 [file IAI.00673-20_s00001.pdf]

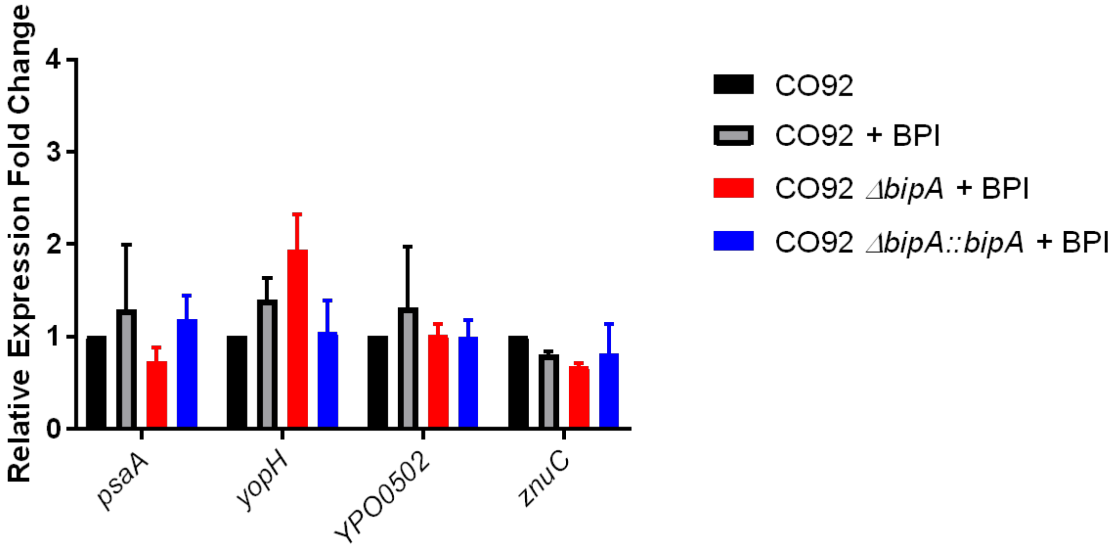

Figure S1: qRT-PCR analysis of genes encoding proteins thought to be regulated by BipA.

Wild type, *ΔbipA*, or *ΔbipA::bipA Y. pestis* CO92 was incubated in PMH2 minimal media with or without 40 mg/mL BPI for 1 hour. RNA was extracted and RT-PCR was performed to determine the relative transcript levels of *psaA*, *yopH*, *YPO0502*, and *znuC*. Error bars represent SD. Significance was calculated using One-Way ANOVA. Data are presented as pool of 3 independent experiments.
