# Supplementary material for: The Yersinia pestis GTPase BipA Promotes Pathogenesis of Primary Pneumonic Plague
Source: Infect Immun. 2021 Jan 19;89(2):e00673-20. doi: 10.1128/IAI.00673-20 (PMC7822129; doi:10.1128/IAI.00673-20)
Supplement: Supplemental file 2 [file IAI.00673-20_s00003.pdf]

Table S1. Putative BipA-Regulated Proteins at Mid-Logarithmic Phase Growth in BHI Medium. List of genes encoding proteins with greater than two-fold change in regulation and at least 10 spectral counts by LC-MS/MS.

Gene	Fold Change (*ΔbipA/* wild type *Y. pestis*)
YPO1258	4
YPO0639	3
mgtB	2.5
hisA	2.5
alr1	2.33
yopH	2.25
YPO0303	2.14
znuC	2.13
YPO0639	2
mltC	2
rlmF	-2.09
YPO0651	-2.14
mreC	-2.14
pst	-2.17
psaA	-2.20
aas	-2.2
ssuE	-2.2
YPO0416	-2.2
YPO2576	-2.25
YPO3308	-2.25
coaD	-2.25
cmr	-2.25

yfeB	-2.4
pabB	-2.5
plsX	-2.6
oppF	-2.67
csy2	-2.71
YPO2745	-2.75
der	-2.76
folD	-2.83
rnfC	-3
YPO0502	-12.48
bipA	Absent from Δ*bipA* *Y. pestis*
