# Supplementary material for: The Yersinia pestis GTPase BipA Promotes Pathogenesis of Primary Pneumonic Plague
Source: Infect Immun. 2021 Jan 19;89(2):e00673-20. doi: 10.1128/IAI.00673-20 (PMC7822129; doi:10.1128/IAI.00673-20)
Supplement: Supplemental file 3 [file IAI.00673-20_s00002.pdf]

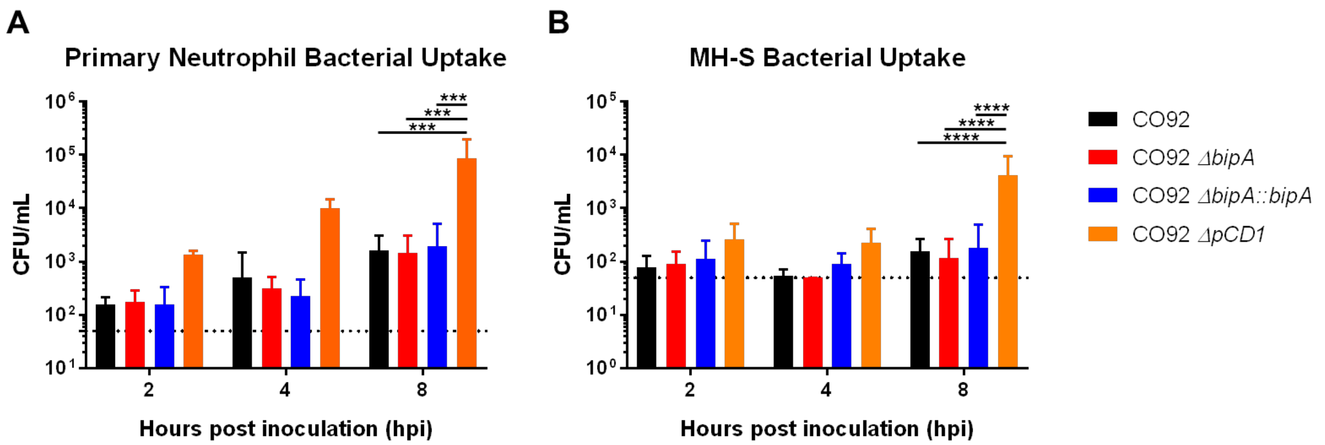

Figure S2: BipA has no role in resistance to phagocytosis by primary human neutrophils or MH-S cells

A) Human primary human neutrophils and B) immortalized murine alveolar macrophages (MH-S) were inoculated with wild type, *ΔbipA*, *ΔbipA::bipA,* or *ΔpCD1 Y. pestis* CO92 using an MOI of 1:1 bacterial to host cell. After indicated time points post-inoculation, cells were treated with gentamicin for 1 hour to kill extracellular bacteria. Cells were lysed and intracellular bacteria were subsequently determined by serial dilution and plating. Dotted line represents limit of detection. Significance was calculated with Two-Way ANOVA with ***p≤.0005 and ****p≤.0001. Error bars represent SD. Data are presented as pool of 3 independent experiments.
