# Supplementary material for: The Yersinia pestis GTPase BipA Promotes Pathogenesis of Primary Pneumonic Plague
Source: Infect Immun. 2021 Jan 19;89(2):e00673-20. doi: 10.1128/IAI.00673-20 (PMC7822129; doi:10.1128/IAI.00673-20)
Supplement: Supplemental file 4 [file IAI.00673-20_s00005.pdf]

Table S3. Resistance of CO92 *Y. pestis*, CO92 *ΔbipA Y. pestis*, and CO92 *ΔbipA::bipA Y. pestis* to antimicrobial peptides

	Minimum Inhibitory Concentration (MIC) μg/mL		
Strain	Polymyxin B	LL-37	BPI
CO92	4	128	>128
CO92 *ΔbipA*	4	128	>128
CO92 *ΔbipA::bipA*	4	128	>128
