# Supplementary material for: The Yersinia pestis GTPase BipA Promotes Pathogenesis of Primary Pneumonic Plague
Source: Infect Immun. 2021 Jan 19;89(2):e00673-20. doi: 10.1128/IAI.00673-20 (PMC7822129; doi:10.1128/IAI.00673-20)
Supplement: Supplemental file 5 [file IAI.00673-20_s00004.pdf]

Table S2. Putative BipA-Regulated Proteins in the Presence of 40 mg/mL BPI. List of genes encoding proteins with greater than two-fold change in regulation and at least 10 spectral counts by LC-MS/MS.

Gene	Fold Change (*ΔbipA/* wild type *Y. pestis*)
znuC	3.5
hdeB	3.45
YPO0130	2.93
malM	2.33
YPO2315	2.25
ubiB	2.2
appR	2.11
yegP	2.08
bfr	2.06
thiI	-2.25
YPO1270	-2.56
aceC	-2.6
aefA	-3
YPO3523	-3
der	-3.24
yadF	-4
yscJ	-5
bisB	-5.5
YPO0500	-6.2
YPO0502	-9.82
metH	-15
YPO1242	-16

YPO3706	-19
YPO0501	-121
bipA	Absent from *ΔbipA* *Y. pestis*
clpB	Absent from *ΔbipA* *Y. pestis*
YPO0102	Absent from *ΔbipA* *Y. pestis*
YPO0498	Absent from *ΔbipA* *Y. pestis*
YPO0987	Absent from *ΔbipA* *Y. pestis*
